# Comparison of the Anti-Inflammatory and Analgesic Effects in Rats of Diclofenac-Sodium, Felbinac and Indomethacin Patches

**Published:** 2011-09

**Authors:** Kozo Takayama, Akihiko Hirose, Ikuko Suda, Atsushi Miyazaki, Masao Oguchi, Masako Onotogi, Grigorios Fotopoulos

**Affiliations:** 1 *Hoshi University, Japan;*; 2 *Novartis Pharma K.K. OTC Buisiness Unit, Japan;*; 3 *Ina Research Inc., Japan;*; 4 *Novartis Consumer Health, Switzerland*

**Keywords:** topical nonsteroidal anti-inflammatory drugs, diclofenac, felbinac, indomethacin, patch, anti-inflammatory effect, analgesic effect, carrageenan-induced paw pad edema, brewer’s yeast-induced hyper algesia

## Abstract

**Background:**

Topically applied nonsteroidal anti-inflammatory drugs (NSAIDs) are used widely for the treatment of pain and inflammation in musculoskeletal disorders. This study compared the analgesic and anti-inflammatory effects of patches of 1% diclofenac-sodium, 3.5% and 0.5% felbinac and 3.75% indomethacin in rats using the carrageenan-induced paw pad edema model and the brewer’s yeast-induced hyper algesia model. Two studies were conducted: in the preliminary study, the patch was removed at 2 or 24 hrs after application, and in the main study the patch was removed at 2 hrs. The volume of the right hind paw and the pain threshold were assessed at 1, 3, 5, and 7 hrs after induction of inflammation in both studies.

**Results:**

In the main study, the edema ratio in the 1% diclofenac group at 5 hrs after induction of inflammation and the AUEC (Area Under the Effect Curve) were significantly lower than in the control animals (*p*=0.009). The edema suppression rate in the 1% diclofenac group (12.1–33.2%) was higher than in the 3.5% and 0.5% felbinac and 3.75% indomethacin groups. The pain threshold ratio did not decrease in the 1% diclofenac group and it was significantly higher than in the control group at 3 (*p*=0.0004) and 5 hrs (*p*=0.029). The 1/AUEC was significantly lower than that in the control group (*p*=0.004) and the lowest among all the NSAID groups.

**Conclusions:**

This study demonstrated that the analgesic and anti-inflammatory effects of the 1% diclofenac sodium patch in a rat model may be exerted more promptly and persistently than with the 3.5% and 0.5% felbinac and 3.75% indomethacin patches.

## BACKGROUND

Diclofenac is a potent nonsteroidal anti-inflammatory drug (NSAID) effective in treating pain and inflammation in various acute and chronic conditions (1). Diclofenac is a phenylacetic acid derivative, usually formulated as a sodium or potassium salt and due to its weak acidic character, high protein binding capacity and low volume of distribution it preferentially targets deep inflamed tissues, such as the joints, where it accumulates up to 20 times more than in plasma (2, 3). As with all NSAIDs, the mechanism of action of diclofenac is considered to be the suppression of prostaglandin (PG) synthesis through inhibition of cyclooxgenease (COX), an enzyme that catalyzes the conversion of arachidonic acid into thromboxane and prostacyclin (4–6).

Oral formulations of diclofenac have been used extensively in clinical practice for over 30 years and have been demonstrated to be effective and generally well tolerated (7). However, use of oral diclofenac may cause systemic side effects (e.g., in the gastrointestinal system) in certain patients, especially if the drug is used at high doses for long periods of time (1). To reduce the potential for systemic side effects, topical formulations of diclofenac were developed to facilitate targeted delivery at the site of pain and inflammation, while reducing systemic absorption and exposure (1). Topical diclofenac formulations have also been clinically proven to be effective in a variety of acute or chronic musculoskeletal conditions such as soft-tissue injuries, rheumatic disorders and osteoarthritis (1, 4–6).

In this study, using the carrageenan-induced rat paw pad edema model and the brewer’s yeast-induced rat hyper algesia model (8–11), we evaluated the anti-inflammatory and analgesic effects of a 1% (15 mg) diclofenac sodium patch. The effect of the diclofenac patch was compared to with commercially available 0.5% or 3.5% felbinac- and 3.75% indomethacin-containing patches.

## METHODS

### Animals

Wistar (WI) male rats obtained from Charles River Laboratories Japan, Inc. were used. The animals were housed for one week (acclimatization period) in stainless steel wire cages (29 cm W × 22 cm D × 21 cm H) under the following conditions: temperature 23–25°C, relative humidity 48–63%, and a 12-hour lighting cycle (lighting hours: 7:00–19:00). Animals were allowed to ingest food and tap water ad libitum during the study. The animal experiments were approved by the “Animal Experiment Review Committee” of Ina Research Inc. on March 9, 2009, and were performed by Ina Research Inc.

### Products Used

In the preliminary study, 1% 15 mg diclofenac sodium patch (1% Voltaren^®^ Patch, Dojin Iyaku-Kako Co., Ltd.) compared with 3.5% felbinac 35 mg patch, (Patex^®^, Nipro Patch Co., Ltd.)

In the main study, 1% (15 mg) diclofenac sodium patch (1% Voltaren^®^ Patch, Dojin Iyaku-Kako Co., Ltd; 7 × 10 cm) was compared with 3.5% (35 mg) felbinac patch (Patex^®^, Nipro Patch Co., Ltd; 7 × 10 cm), 0.5% (10 mg) felbinac patch (Feitas^®^ L, Yutoku Pharmaceutical Ind. Co., Ltd; 7 × 10 cm), and 3.75% indomethacin patch (Inside^®^ Patch, SSP Co., Ltd; 7 × 10 cm). Table 1 describes the amount of active ingredient contained per piece of patch applied to the rats.

**Table 1:** Pharmaceutical preparations used in this study

Preparation	Content (mg/2.0 cm × 1.75 cm)

1% Diclofenac (Voltaren^®^) patch	0.75
3.5% Felbinac patch	1.75
0.5% Felbinac patch	0.25
3.75% Indomethacin patch	1.31^a^

a Calculated using the quantity of the active pharmaceutical ingredient given under “Composition” in the package insert.

### Study Design

A preliminary and a main study were performed.

#### • Preliminary Study

**Anti-Inflammatory Study.** The volume of the right hind footpad paw of Wistar male rats (aged 5 weeks) was measured at baseline with a paw volume-measuring instrument (7140, LMS Co., Ltd.), and 24 randomly selected rats were assigned to 4 groups (6 animals per group).

In each study, a piece of patch was applied at the center of the dorsum of the right hind footpad paw and fixed with Band-Aid. During the treatment period an Elisabeth collar was put on each rat to avoid patch removal by the animal. The patch was removed at 2 hours or 24 hours after application, and 0.1 mL of an aqueous 1 w/v% suspension of λ-carrageenan (Sigma-Aldrich Co., Lot No. WA14463) in physiological saline was injected subcutaneously into the right hind footpad paw pad as an inflammation-inducing agent. At 1, 3, 5, and 7 hours after the induction of inflammation, the paw volume was measured, and the edema rate (%) was calculated using the baseline value according to the following formula.

Edema rate%=Paw volume at each measuring timer point−Baseline Paw volume mLBaseline Paw volume mL×100

**Analgesic Study.** Increasing pressure was applied at the center of the dorsum of the right hind footpad paw of Wistar male rats (aged 7 weeks) using a Pain Pressure Stimulation Device (MK-300, Muromachi Kikai Co., Ltd.). Pressure was applied twice on the day of grouping to determine the pain threshold value defined as the minimum pressure causing a pain reaction, such as crying or struggling to escape. Twenty-four rats whose average of two measured pain threshold values was close to the entire average value were randomly selected and assigned to 4 groups (6 animals in each group). The second pain threshold value of each rat was used as the baseline value.

Each study patch piece was applied at the center of the dorsum of the right hind footpad paw of each rat and fixed with Band-Aid and an Elisabeth collar was applied. The study patch was removed 2 hours or 24 hours after application, and 0.1 mL of an aqueous 10 w/v% suspension of dried brewer’s yeast (Asahi Food & Healthcare Co., Ltd.; Lot No. 901191) in physiological saline was injected subcutaneously into the right hind footpad paw. At 1, 3, 5, and 7 hours after the induction of inflammation, pain values were measured.

The pain threshold value ratio was calculated using the baseline pain threshold value according to the following formula:

Pain threshold value ratio=Pain threshold value at each measuring time point gBaseline pain threshold value g

#### • Main Study

**Anti-Inflammatory Study.** Wistar male rats (aged 5 weeks) were measured for the volume of the right hind footpad paw in advance with the paw volume-measuring instrument, and 50 randomly selected rats were assigned to 5 groups (10 animals in each group). The measured paw volumes were handled as the baseline values.

In each study, a piece of drug patch was applied at the back center of the right hind footpad paw of each rat and fixed with Band-Aid. During the treatment, an Elisabeth collar was put on each rat so that the drug patch would not be removed by the rat. The drug patch was removed at 2 hours after application, and 0.1 mL of an aqueous 1 w/v% suspension of λ-carrageenan in physiological saline was injected subcutaneously into the right hind footpad paw pad as an inflammation-inducing agent. In the inflammation control group, no drug patch was applied, and only the inflammation-inducing agent was administered. At 1, 3, 5, and 7 hours after the induction of inflammation, the paw volume was measured, and the edema rate (%) was calculated using the baseline value.

In addition, from the edema rate data at individual measuring time points, the edema suppression rate (%) and the area under the effective curve (AUEC) were calculated as follows:

Edema suppression rate%=Edema rate of inflammation control group−Edema rate of study drug group %Edema rate of inflammation control group %×100

AUEC=1/2 (3a + 4b + 4c + 2d)

- Edema rate at 1 hour after induction of inflammation
- Edema rate at 3 hours after induction of inflammation
- Edema rate at 5 hours after induction of inflammation
- Edema rate at 7 hours after induction of inflammation

**Analgesic Study.** Wistar male rats (aged 7 weeks) were given an increasing pressure at the back center of the right hind footpad paw with the pressure pain-measuring instrument twice on the day of grouping to measure the pain threshold value. Fifty rats whose average of two measured pain threshold values was close to the entire average value were selected and randomly assigned to 5 groups (10 animals in each group). The second pain threshold value of each rat was handled as the baseline value.

In each study, the drug patch was applied at the back center of the right hind footpad paw and fixed with Band-Aid. During the treatment, an Elisabeth collar was put on each rat so that the drug patch would not be removed by the rat. The drug patch was removed at 2 hours after application, and 0.1 mL of an aqueous 10 w/v% suspension of dried brewer’s yeast in physiological saline was injected subcutaneously into the right hind paw pad as an inflammation-inducing agent. In the analgesia control group, no study drug was applied, and only the inflammation-inducing agent was administered. At 1, 3, 5, and 7 hours after the induction of inflammation, the pain threshold value was measured.

The pain threshold value ratio was calculated using the baseline pain threshold value.

In addition, from the pain threshold value ratios observed at 1, 3, 5, and 7 hours after induction of inflammation, the reciprocal value of the area under the effective curve (AUEC) was calculated according to the following formula:

1/AUEC=2/ (1 + 3a + 4b + 4c + 2d)

- Pain threshold value ratio at 1 hour after induction of inflammation
- Pain threshold value ratio at 3 hours after induction of inflammation
- Pain threshold value ratio at 5 hours after induction of inflammation
- Pain threshold value ratio at 7 hours after induction of inflammation

### Statistical Processing

The mean and standard deviation for each group were calculated for edema rate, AUEC, pain threshold value ratio, and 1/AUEC. Variance uniformity was tested by the Bartlett method for edema rate, AUEC, pain threshold value ratio, and 1/AUEC (significance level: 5%). In cases where the variance was uniform, the difference in mean value was tested by the Dunnett method between the inflammation control group and each of the other 4 groups or between the 1% diclofenac patch group and each of the other study drug treatment groups. In cases where the variance was not uniform, the difference in average rank was tested by the Dunnett method between the inflammation control group and each of the other 4 groups or between the 1% diclofenac patch group and each of the other study drug treatment groups. The significance level in testing was set at two-sided 5%. When a significant difference was seen between the inflammation control group and the 1% diclofenac patch group “*p*<5%” or “*p*<1%” was shown in the figures and tables.

## RESULTS

### Preliminary Study

Application of the study patch for 24 hours before induction of inflammation, resulted in maximum edema rate (40.8 ± 22.8%) in the 1% diclofenac patch group at 3 hours after induction of inflammation vs 5 hours (35.2 ± 4.4%) in the 3.5% felbinac patch group. When the study patch was applied for 2 hours before induction of inflammation, maximum edema rate was observed at 5 hours after induction of inflammation in both the 1% diclofenac patch (36.4 ± 7.9%) and the 3.5% felbinac patch groups (43.2 ± 10.5%).

Application of the patch for 24 hours before induction of inflammation, resulted in a decrease of the pain threshold ratio in a time-dependent manner with a minimum at 7 hours after induction of inflammation in the 1% diclofenac patch group (0.74 ± 0.20) and at 5 hours (0.74 ± 0.08) in the 3.5% felbinac patch group. When the patches were applied for 2 hours before induction of inflammation, a minimum pain threshold ratio was demonstrated at 5 hours after induction of inflammation in the 1% diclofenac patch group (0.78 ± 0.22) and at 7 hours in the 3.5% felbinac patch group (0.72 ± 0.09).

The preliminary study demonstrated no differences between the 2 hours and 24 hours application. Therefore, the main study was performed by applying each patch for 2 hours before the induction of inflammation.

### Main Study

**Figure 1:**
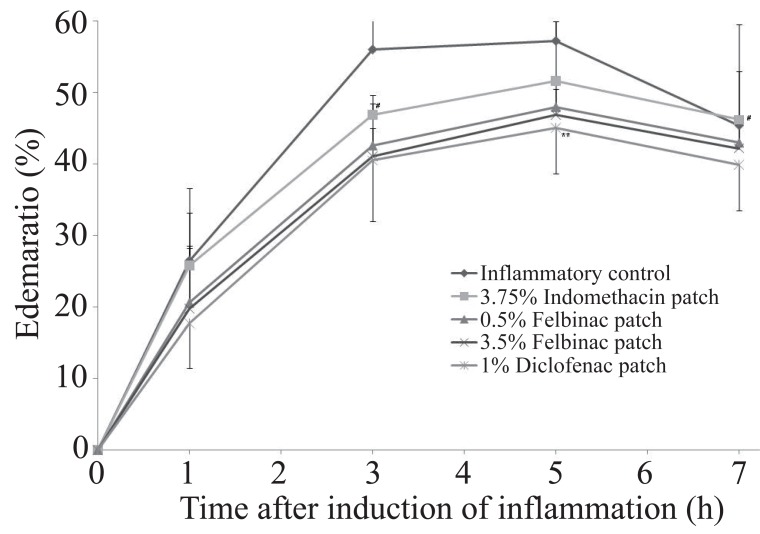
Time course of the edema ratio after topical application of 1% diclofenac patch or other NSAID patch on the carrageenan-induced hind footpad paw edema in rats. Data are expressed as the mean ±S.D. of 10 animals. **p<0.01: 1% diclofenac patch: significantly different from the inflammatory control group; #*p*<0.05: 1% diclofenac patch: significantly different from the 3.75% indomethacin patch group (Dunnett’s test).

**Anti-inflammatory Study.** In the control group, the edema rate increased with time after induction of inflammation with a maximum at 5 hours (Figure 1). The AUEC was 311.5 ± 98.5 (Table 2).

**Table 2:** Anti-inflammatory effects of 1% diclofenac patch and other NSAID patches on carrageenan induced edema at hind footpad paw in rats

	Group	Number of animals	After induction of inflammation				
			1h	3h	5h	7h	AUEC

Edema ratio (%)	Inflammatory control	10	26.5 ± 10.1	56.0 ± 23.3	57.2 ± 15.5	45.4 ± 14.1	311.5 ± 98.5
	3.5% Felbinac patch	10	19.8 ± 8.8	41.1 ± 3.9	46.9 ± 3.6	42.2 ± 3.3	247.9 ± 20.1^a^
	0.5% Felbinac patch	10	20.6 ± 7.6	42.6 ± 5.9	48.0 ± 4.3	43.0 ± 3.6	255.2 ± 26.3
	3.75% Indomethacin patch	10	25.8 ± 7.4	46.9 ± 2.7^c^	51.6 ± 8.4	46.2 ± 6.8^c^	281.7 ± 30.4^d^
	1% Diclofenac patch	10	17.7 ± 6.3	40.5 ± 8.5	45.1 ± 6.4^b^	39.9 ± 6.4	237.7 ± 40.1^b^

Each value represents the mean ± S.D. of 10 animals. Significantly different from the inflammatory control group.

a *p*<0.05,

b *p*<0.01 (Dunnett’s test). Significantly different from 1% diclofenac patch group.

c *p*<0.05,

d *p*<0.01 (Dunnett’s test).

**Figure 2:**
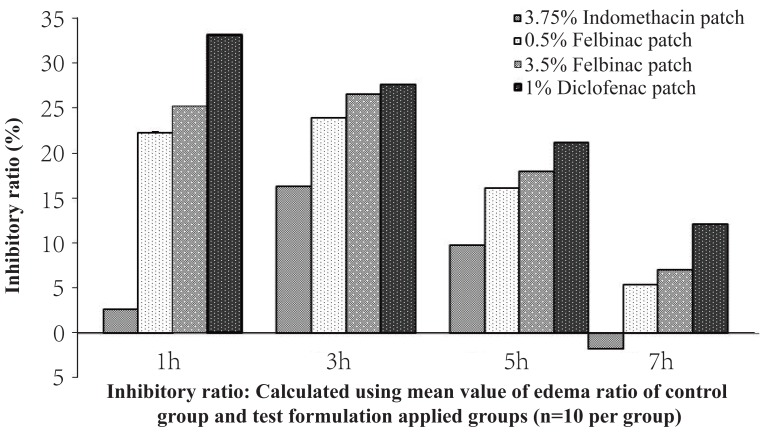
Time course of inhibitory ratio after topical application of 1% diclofenac patch or other NSAID patch on the carrageenan-induced hind footpad paw edema in rats, Inhibitory ratio calculated using mean value of edema ratio of inflammatory control and test formulation applied groups.

**Figure 3:**
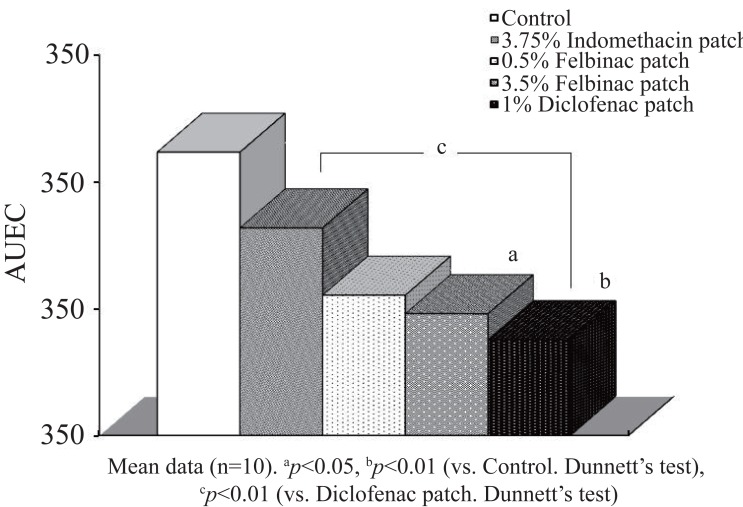
Anti-inflammatory effect (AUEC: Area under the effect curve) of 1% diclofenac patch or other NSAID patch on the carrageenan-induced hind footpad paw edema in rats. Data are expressed as the mean ± S.D. of 10 animals.

In the 1% diclofenac patch group, the edema rates were lower than those in the inflammation control group as well as in the other study groups from 1 hour after induction of inflammation, and a significant difference was seen between the 1% diclofenac patch group and the inflammation control group (*p*=0.009) at 5 hours after induction of inflammation. The edema suppression rate ranged from 12.1% to 33.2% and was higher in the 1% diclofenac patch group than in the 3.5% and 0.5% felbinac and 3.75% indomethacin groups (Figure 2), while the AUEC was 237.7 ± 40.1, being significantly lower than that in the inflammation control group (*p*=0.009) (Figure 3).

In the 3.5% felbinac patch group, the edema rates were lower than those in the control group, but no significant difference was seen at any time point. The AUEC (247.9 ± 20.1) was significantly lower than that in the control group (*p*=0.017). When compared with the 1% diclofenac patch group there were no significant differences in edema rate and AUEC at any time point. The edema suppression rate ranged from 7.0% to 26.6% and was lower than in the 1% diclofenac patch group.

In the 0.5% felbinac patch group, the edema rates remained lower than those in the control group, but no significant differences in edema rate and AUEC were seen at any time point in comparison with the control group or the 1% diclofenac patch group. The edema suppression rate ranged from 5.3% to 23.9% and was lower in the 0.5% felbinac patch group than in the 1% diclofenac patch group and the 3.5% felbinac patch group.

In the 3.75% indomethacin patch group, the edema rates were lower than those in the control group until 5 hours after induction of inflammation, but no significant differences in edema rate or AUEC were seen at any time point in comparison with the control group. When compared with the 1% diclofenac patch group, the edema rates at 3 (46.9 ± 2.7%) and 7 hours (46.2 ± 6.8%) after induction of inflammation, as well as the AUEC (281.7 ± 30.4), were significantly higher than those in the 1% diclofenac patch group [edema rate: *p*=0.018 (3 hours), *p*=0.031 (7 hours), AUEC: *p*=0.007]. The edema suppression rate ranged from -1.8% to 16.3% and was lower in the 3.75% indomethacin patch group than in the 1% diclofenac patch group.

**Figure 4:**
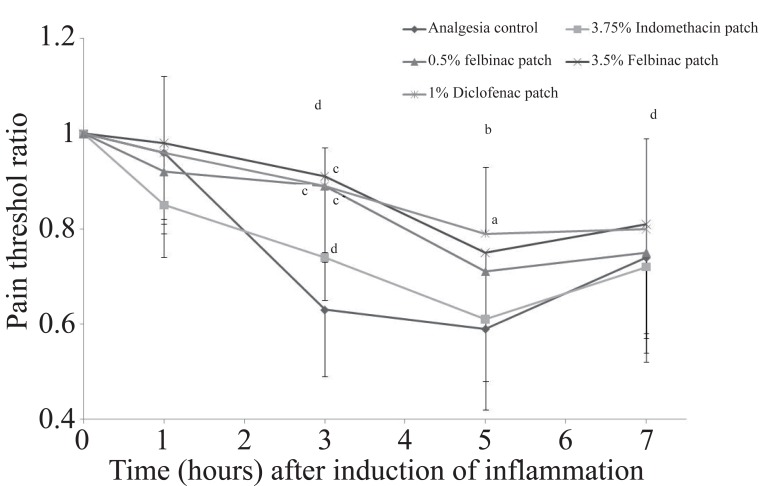
Time course of changes in pain threshold after topical application of 1% diclofenac patch or other NSAID patch on the pain threshold induced by 10% brewer’s yeast suspension injection in rat hind footpad paw. Data are expressed as the mean ± S.D. of animals. **p<0.01, ***p<0.001: Significantly different from the analgesia control group; #*p*<0.05: Significantly different from the indomethacin patch group. (Dunnett’s test).

**Study on Analgesic Effect.** In the control group, the pain threshold ratio decreased with time with a minimum at 5 hours after induction of inflammation (Figure 4). The 1/AUEC was 0.200 ± 0.028 (Table 3).

**Table 3:** Analgesic effect of 1% diclofenac patch and other NSAID patches on brewer’s yeast induced at hind footpad paw in rats

	Group	Number of animals	After induction of inflammation				
			1h	3h	5h	7h	1/AUEC

Pain threshold ratio	Analgesia control	10	0.96 ± 0.15	0.63 ± 0.14	0.59 ± 0.17	0.74 ± 0.20	0.200 ± 0.028
	3.5% Felbinac patch	10	0.98 ± 0.16	0.91 ± 0.18^c^	0.75 ± 0.13	0.81 ± 0.24	0.166 ± 0.020^b^
	0.5% Felbinac patch	10	0.92 ± 0.13	0.89 ± 0.14^c^	0.71 ± 0.23	0.75 ± 0.23	0.175 ± 0.024^a^
	3.75% Indomethacin patch	10	0.85 ± 0.11	0.74 ± 0.09^d^	0.61 ± 0.13	0.72 ± 0.14	0.193 ± 0.018^e^
	1% Diclofenac patch	10	0.96 ± 0.16	0.89 ± 0.08^c^	0.79 ± 0.14^a^	0.80 ± 0.19	0.165 ± 0.016^b^

Each value represents Mean ± S.D. of 10. Significantly different from analgesia control group.

a *p*<0.05,

b *p*<0.01,

c *p*<0.001 (Dunnett’s test). Significantly different from 1% diclofenac patch group.

d *p*<0.05,

e *p*<0.01 (Dunnett’s test).

**Figure 5:**
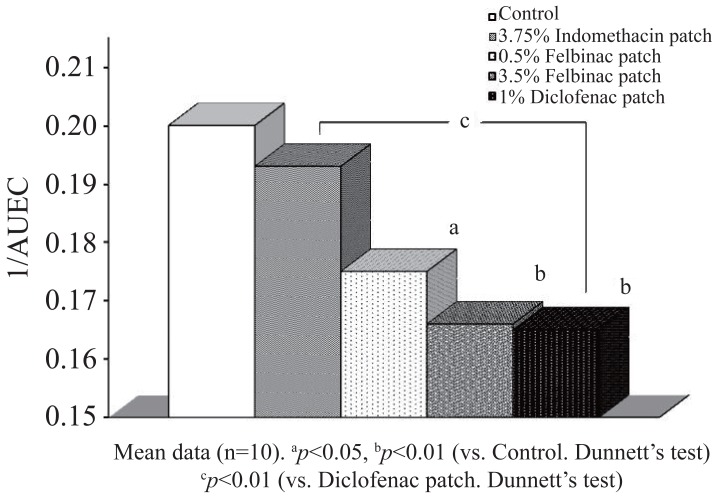
Analgesic effect (1/AUEC: Reciprocal of Area under the effect curve) of 1% diclofenac patch or other NSAID patch on brewer’s yeast induced at hind footpad paw in rats. Data are expressed as the mean ± S.D. of 10 animals.

The pain threshold ratio did not decrease in the 1% diclofenac patch group, but it was significantly higher than in the control group at 3 (0.89 ± 0.08, *p*=0.0004) and 5 hours (0.79 ± 0.14, *p*=0.029) after induction of inflammation. The 1/AUEC (0.165 ± 0.016) was significantly lower than that in the control group (*p*=0.004) and the lowest among all the groups (Figure 5).

In the 3.5% felbinac patch group the pain threshold ratio did not decrease and was significantly higher than that in the control group at 3 hours after induction of inflammation (0.91 ± 0.18, *p*=0.0001). The 1/AUEC (0.166 ± 0.020) was also significantly lower than that in the control group (*p*=0.004). When compared with the 1% diclofenac patch group, no significant differences were observed in pain threshold ratio or 1/AUEC at any time point.

The pain threshold ratio did not decrease in the 0.5% felbinac patch group and was significantly higher than in the control group at 3 hours after induction of inflammation. The 1/AUEC (0.175 ± 0.024) was significantly lower than that in the control group (*p*=0.0003), while no significant differences were observed compared to the 1% diclofenac patch group for both pain threshold ratio and 1/AUEC at any time point.

Finally, in the 3.75% indomethacin patch group, the pain threshold ratio did not decrease and no significant differences were seen in pain threshold ratio or 1/AUEC at any time point in comparison with the control group. When compared with the 1% diclofenac patch group the pain threshold value ratio at 3 hours after induction of inflammation was marginally lower (0.74 ± 0.09, *p*=0.046), and the 1/AUEC was significantly higher (0.193 ± 0.018, *p*=0.009).

## DISCUSSION

In this study, the anti-inflammatory and analgesic effects of 1% diclofenac-sodium 15 mg patch (Voltaren^®^ patch 1%) were assessed and compared with those of the 3.5% or 0.5% felbinac and 3.75% indomethacin patches. To evaluate the anti-inflammatory effects, carrageenan-induced acute rat paw edema was used as an acute inflammatory model, which is commonly used (12). In parallel, to evaluate the analgesic effect, brewer’s yeast-induced rat paw hyperalgesia was used as an inflammatory algesia model.

The results of this study demonstrated that the induced edema was effectively reduced by the 1% diclofenac patch, of which the magnitude of effect was higher than that of the 3.5% or 0.5% felbinac and 3.75% indomethacin patches at the time points measured (1 hour to 7 hours after induction of inflammation). Furthermore, the AUEC up to 7 hours after induction of inflammation was the lowest in the 1% diclofenac patch group among all the patch groups.

The edema reduction rate was higher in the 1% diclofenac patch group than in the 0.5% and the 3.5% felbinac groups as well as in the 3.75% indomethacin group at 1 hour and 7 hours after induction of inflammation. These *in vivo* results corroborate the anti-inflammatory properties of the 1% diclofenac patch and suggest that it would exert such effects more promptly and more persistently than the other NSAID patch preparations tested.

Furthermore, it was demonstrated that the decrease in the pain threshold ratio was suppressed already at 1 hour after induction of inflammation in the 1% diclofenac patch group. The suppression was similar or higher (at 5 hours) when compared to the 3.5% felbinac group. Significantly also, the 1% diclofenac patch group’s 1/AUEC value (up to 7 hours) was the lowest among the all groups.

In this study, no pharmacokinetic parameters were evaluated, but the results obtained in this animal model suggested that diclofenac is adequately delivered to the inflammation site to exert its anti-inflammatory and analgesic effects, which is in agreement with other studies demonstrating the preferential distribution of topically applied diclofenac at the inflamed site (1–3).

Remarkably, in the present study, the 1% diclofenac patch demonstrated a stronger anti-inflammatory and analgesic effect when compared to felbinac and indomethacin patches. A significant difference was observed between the 1% diclofenac-sodium patch and the 3.75% indomethacin patch with regard to the AUEC and the 1/AUEC. In this study, the reasons for this difference have not been elucidated. It is generally accepted that diclofenac preferentially distributes to the inflammation site and exhibits superior potency in inhibiting PGE2 production when compared with indomethacin and other NSAIDs (13). It is also known that the pH in damaged, inflamed tissues is lowered due to acidic substances that are released from the stimulated cells and migrating leukocytes in association with direct activation of the sensory neuron cations involved in pain sensation. It has been reported that the transcription of acid-sensing ion channel (ASIC) of sensory neurons is increased at the inflammation site *in vivo*, since the inflammation-inducing mediators, such as NGF, serotonin, interleukin-1, and bradykinin, etc., are released due to the intra-tissue acidosis (14). There are four ASIC isoforms (ASIC1a, ASIC1b, ASIC2b, and ASIC3) and Voilley *et al*. (15) reported that diclofenac inhibited the activity of ASIC3, while indomethacin did not inhibit the activity of any isoforms. This may explain why the 1% diclofenac patch demonstrated a more potent anti-inflammatory and analgesic effect than the 3.75% indomethacin patch in this study. Finally, the 1% diclofenac patch contains l-menthol and N-methyl-2-pyrrolidone as absorption-promoting agents. The formulation has been optimized so that these two components modify the intercellular lipid status among the corneocytes and enhance permeation of diclofenac into the subcutaneous region (16), which may also contribute to the superior effect of this diclofenac patch formulation.

## CONCLUSION

Overall, this study, utilizing a well established animal model of pain and inflammation, confirms the clinically proven analgesic and anti-inflammatory effects of topically applied diclofenac, using a commercial patch formulation. In addition, these results suggest that 1% diclofenac-sodium 15 mg patch is at least comparable to 0.5% and 3.5% felbinac patches and superior to 3.75% indomethacin patch in terms of analgesic and anti-inflammatory potential, in the animal model tested. While these data provide a clear indication of the efficacy of these formulations and suggest that 1% diclofenac-sodium patch would be a useful formulation in clinical practice, comparative double blind randomized clinical studies are required to demonstrate its efficacy.

## AUTHOR’S CONTRIBUTIONS

KT approved the design of the study and helped to draft the manuscript. AH and IS participated in the design of the study and helped to draft the manuscript. AM, MO, and MO participated in the execution of the study. GF approved the design of the study and helped to draft the manuscript. All authors read and approved the final manuscript.

## References

[1] Zacher J, Altman R, Bellamy N, Brühlmann P (2008). Topical diclofenac and its role in pain and inflammation: an evidence-based review. Curr. Med. Res. Opin.

[2] Schweitzer A, Hasler-Nguyen N, Zijlstra J (2009). Preferential uptake of the non steroid anti-inflammatory drug diclofenac into inflamed tissues after a single oral dose in rats. BMC Pharmacol.

[3] Brune K (2007). Persistence of NSAIDs at effect sites and rapid disappearance form side-effect compartments contributes to tolerability. Curr. Med. Res. Opinion.

[4] Mitchell JA, Warner TD (1999). Cyclo-oxygenase-2: pharmacology, physiology, biochemistry and relevance to NSAID therapy. Br. J. Pharmacol.

[5] Vane J (2003). The mechanism of action of anti-inflammatory drugs. Int. J. Clin. Pract.

[6] Vane J (2000). Aspirin and other anti-inflammatory drugs. Thorax.

[7] Collins SL, Moore RA, McQuay HJ, Wiffen PJ (2000). Single dose oral ibuprofen and diclofenac for postoperative pain. Cochrane Database Syst. Rev.

[8] Takashima T, Kado Y, Ono R, Kumada S (1972). Anti-inflammatory effect of GP45840. Kiso to Rinsyo.

[9] Tsurumi K, Hiramatsu Y, Nozaki M, Hayashi M (1973). Anti-inflammatory effects of N-(2,6-dicholorophenyl)-o-aminophenylacetic acid and its sodium salt and N-(2, -dichlorophenyl)-anthranilic acid and its sodium salt. Report 1: Acute inflammation. J. Jap. Pharmacol. Sci.

[10] Tsurumi K, Hiramatsu Y, Yamaguchi A, Hayashi M (1973). Anti-inflammatory effects of N-(2,6-dicholorophenyl)-o-aminophenylacetic acid and its sodium salt and N-(2, -dichlorophenyl)-anthranilic acid and its sodium salt. Report 2: Acute inflammation. J. Jap. Pharmacol. Sci.

[11] Aoki R (1972). A new anti-inflammatory drug-screening method using skin reactive factor (SRF) -investigation of anti-inflammatory effect of GP45840-. Kiso to Rinsyo.

[12] Saito H, Nomura Y (1990). Iyakuhin no kaihatsu.

[13] Schunack W (1988). Gastrointestinale Nebenwirkungen nichtsteroidaler Antirheumatika. Z. Allg. Med.

[14] Voilley N, de Weille J, Mamet J, Lazdunski M (2001). Nonsteroid anti-inflammatory drugs inhibit both the activity and the inflammation-induced expression of acid-sensing ion channels in nociceptors. J. Neurosci.

[15] Voilley N (2004). Acid-Sensing ion channels (ASICs): New targets for the analgesic effects of non-steroid anti-inflammatory drugs (NSAIDs). Curr. Drug Targets Inflamm. Allergy.

[16] Obata Y, Takayama K (2003). Front line of transdermal absorption-promoting agents. Journal of Tokyo Hospital Pharmacists Association.

